# The Asymmetric Cell Division Regulators Par3, Scribble and Pins/Gpsm2 Are Not Essential for Erythroid Development or Enucleation

**DOI:** 10.1371/journal.pone.0170295

**Published:** 2017-01-17

**Authors:** Christina B. Wölwer, Nathan Gödde, Luke B. Pase, Imogen A. Elsum, Krystle Y. B. Lim, Faruk Sacirbegovic, Carl R. Walkley, Sarah Ellis, Shigeo Ohno, Fumio Matsuzaki, Sarah M. Russell, Patrick O. Humbert

**Affiliations:** 1 Cell Cycle and Cancer Genetics, Peter MacCallum Cancer Centre, East Melbourne, Australia; 2 La Trobe Institute for Molecular Science, Department of Biochemistry and Genetics, La Trobe University, Melbourne, Australia; 3 Immune Signaling Laboratory, Peter MacCallum Cancer Centre, East Melbourne, Australia; 4 Department of Pathology, University of Melbourne, Parkville, Victoria, Australia; 5 St. Vincent’s Institute of Medical Research, Fitzroy, Victoria, Australia; 6 Department of Medicine, St. Vincent’s Hospital, The University of Melbourne, Fitzroy, Victoria; 7 Department of Molecular Biology, Yokohama City University Graduate School of Medical Science, Yokohama, Japan; 8 Laboratory for Cell Asymmetry, RIKEN Center for Developmental Biology, Kobe, Japan; 9 Sir Peter MacCallum Department of Oncology, University of Melbourne, Melbourne, Australia; 10 Centre for Micro-Photonics, Faculty of Engineering and Industrial Sciences, Swinburne University of Technology, Hawthorn, Australia; 11 Department of Biochemistry and Molecular Biology, University of Melbourne, Parkville, Victoria, Australia; Duke-NUS Graduate Medical School, SINGAPORE

## Abstract

Erythroid enucleation is the process by which the future red blood cell disposes of its nucleus prior to entering the blood stream. This key event during red blood cell development has been likened to an asymmetric cell division (ACD), by which the enucleating erythroblast divides into two very different daughter cells of alternate molecular composition, a nucleated cell that will be removed by associated macrophages, and the reticulocyte that will mature to the definitive erythrocyte. Here we investigated gene expression of members of the Par, Scribble and Pins/Gpsm2 asymmetric cell division complexes in erythroid cells, and functionally tested their role in erythroid enucleation *in vivo* and *ex vivo*. Despite their roles in regulating ACD in other contexts, we found that these polarity regulators are not essential for erythroid enucleation, nor for erythroid development *in vivo*. Together our results put into question a role for cell polarity and asymmetric cell division in erythroid enucleation.

## Introduction

Erythroid enucleation embodies many features of an asymmetric cell division[1–10] and results in the generation of two unique daughter cells: the pyrenocyte containing the condensed nucleus and the anucleated reticulocyte that will further mature into the erythrocyte found in the peripheral blood. One of the first visible steps of establishing polarity prior to nuclear extrusion is the phosphoinositide 3-kinase/microtubule -directed migration of the nucleus to one side of the cytoplasm, whereby the nucleus apposes the cell membrane[5]. This event is followed by a polarized enrichment of cytoskeletal elements, including actin, and myosin II to the future restriction site between pyrenocyte and future red blood cell[6–9]. During the enucleation process specific membrane proteins are either sorted to the nucleus (e.g. Emp protein) or to the future reticulocyte (e.g. spectrin)[7, 10].

Asymmetric cell division (ACD) is crucial for generating cell progeny with diverse functions and requires a polarized distribution of cell fate determinants, cytoskeletal regulators and polarity proteins. The pathways regulating asymmetric cell division have mainly been derived from studies in *Drosophila melanogaster*, particularly from neuroblasts, which undergo up to 20 rounds of asymmetric cell divisions to generate neurons of the larval nervous system[11]. Three cell polarity complexes have been identified to contribute to the establishment of asymmetry in *Drosophila* neuroblasts: the Par, Pins and Scribble complexes[12]. The Pins complex (comprised of Inscuteable (Insc), Partner of Insc (Pins) and a Gαi subunit) regulates mitotic spindle orientation by providing an attachment site for astral microtubules[13]. The Par complex (made up of Par3, Par6 and atypical Protein Kinase C (aPKC)) and the Scribble complex (comprised of Scribble, Discs Large (Dlg) and Lethal Giant Larvae (Lgl)) are required for the establishment of cell polarity that is critical for ACD[14]. All of these complexes are conserved in vertebrates and are thought to play similar roles. For example, Par3 has been implicated in determining polarity in mammalian oogenesis[15], an example of an asymmetric cell division, in which the egg divides to produce an oocyte and a small polar body. Cells of the hematopoietic systems make fate decisions in order to either self-renew, differentiate, proliferate or to undergo apoptosis. ACD may control these decisions[16]. For example, it has been suggested that T-cell development displays many features characteristic of ACD[17]. In line with these observations, studies using knockdown and knockout approaches implicated important roles for the Scribble and Par3 complexes in the development and function of lymphocytes[18]. Pins, also known as Gpsm2 (G protein signaling modulator 2) is thought to enhance haematopoietic stem cell function through altered asymmetric and symmetric divisions[19].

Despite the proposal that erythroid enucleation embodies many features of ACD, the requirement for ACD regulators in this event has not been investigated to date. Given that ACD utilizes a conserved molecular toolbox across species and within different forms of tissue development[20], we examined expression of known critical asymmetric division genes, from the Scribble, Par and Pins complex in orthochromatic erythroblasts, and used the corresponding mouse models to functionally test the role of ACD regulators in erythroid development and enucleation.

## Materials and Methods

### Materials

Phenylhydrazine hydrochloride (PHZ) was purchased from Aldrich Chemistry. PE-Cy7 conjugated anti-CD44 mouse antibodies were purchased from BD Pharmingen. Alexa Fluor 647 conjugated anti-Ter119 mouse antibodies were purchased from Biolegend. Hoechst 33342 was purchased from Invitrogen. Propidium iodide (PI) was purchased from Merck. Rapid Diff stain was purchased from Australian Biostain.

### Animal experiments

This study was carried out in strict accordance with the recommendations of the Victorian Bureau of Animal Welfare, Department of Primary Industries, and the National Health and Medical Research Council's Australian code of practice for the care and use of animals for scientific purposes. The protocol was approved by the Institutional Animal Care and Use Committee: Peter MacCallum Cancer Centre Animal Experimentation Ethics Committee under Permit number E535. All efforts were made to minimize suffering. All mice (71 females, 72 males) used in this study were on a C57BL/6 background and 6–12 weeks of age. Mice were kept at 21°C, with a humidity of approximately 60% on a 14h light/10h dark cycle and fed with standard mouse cubes (Ridley Agri). EpoR-Cre ^ki/+^ mice[21] (referred to as *EpoR-Cre*^+^ in this manuscript) were provided by Dr. Carl Walkley (St. Vincent’s Institute, Melbourne, Australia). Conditional Par3 knockout mice (*Pard3*^fl^) were generated by the laboratory of Prof. Shigeo Ohno (Yokohama City University, Yokohama, Japan)[22]. Conditional Scrib knockout mouse allele (*Scrib*^fl^) were generated in-house[23]. Gpsm2^ΔC^ mice were generated by Fumio Matsuzaki[24]. To induce stress erythropoiesis mice at 6–12 weeks of age were administered intraperitoneal injections of phenylhydrazine hydrochloride (60μg/g) on day 0 and day 1 of the experiments. On day 4, cells were isolated from bone marrow and spleens. Following phenylhydrazine hydrochloride treatment, mice were monitored for signs of discomfort every two hours as defined by lethargy, ruffled fur or a hunched appearance, at which time the mice were considered to have reached the ethically permitted humane endpoint criteria and were humanely euthanized using cervical dislocation.

### FACS analysis of erythropoiesis and enucleation

For analysis of erythropoiesis cells were harvested from bone marrow or spleen and stained for Ter119 and CD44[25]. PI was used to exclude dead cells from the sort. Erythropoiesis was analyzed using FACS LSR II. 20000 viable cells were analyzed for each sample.

Analysis of enucleation was done as previously described[26]. Shortly, *ex vivo* cells isolated from bone marrow or spleen were stained for CD44, Ter119, Hoechst and PI. All Hoechst negative (enucleated) cells were excluded from the sort. Orthochromatic erythroblasts were isolated based on their Ter119 and CD44 expression by FACS Aria II special order system (BD) using the FACS Diva software (BD). Enucleation was analyzed 5h post sort using FACS LSR II. 5000 cells were analyzed per sample. Net percentage of enucleation was then derived by dividing the number of enucleated cells (Ter119^+^/Hoechst^-^) by the sum of enucleated cells and erythroblasts (Ter119^+^/Hoechst^+^), and by subsequently multiplying the quotient by 100.

### RNA isolation, cDNA synthesis and qPCR

Total RNA was extracted from FACS sorted erythroid cells, and reversed transcribed with Superscript III (Invitrogen). Real-time PCR amplification of cDNA was done in triplicates in a StepOnePlus Real-Time PCR system (Applied Biosystems, Carlsbad, CA) using the SYBER Green gene expression assay (Applied Biosystems, USA). All samples were normalized to β2Macroglobulin (β2M) control and fold change between samples was calculated using the comparative C(T) method. Primers used for qRT-PCR are listed below (Table 1).

**Table 1:** Sequences of primers used for QRTPCR.

Target	Forward sequence (5’-3’)	Reverse sequence (5’-3’)
β*2M*	TTCACCCCCACTGAGACT	GTCTTGGGCTCGGCCATA
*scribble*	GAGGAGATTTACCGCTACAGTCGTA	TGTCACTGAGGCCCAACTTTC
*lgl1*	TGTTGTACGCTGCCTCTACTTTG	GGTGCCCGCCCACAT
*lgl2*	GGTGGATTCAACCAAAGCCAA	TTGGCTCTTTGAGTTCCTGACG
*dlg1*	GAGAGTGACGAAGTCGGAGTGA	AATCGGGCTCGTTCCTTCTT
*dlg3*	GGTGGAAAAGAAAGAGCGAGCT	GCATCCTCTTGTCCTAGGT
*dlg4*	CCCAACATGGACTGTCTCTGTATAGT	GGGCGTGTCTTCATCTTGGT
*pins*	GGCCAGTTTCAGTAATTTCA	AGGCTAAAGAAGTCCTCATCCG
*insc*	GTTCGGCTCAGCTGTATGTCTC	GGTAAGTGCACACAGTGTGCCT
*Apkcζ*	AGCCAACGGCCACCTCTT	CAGTACGCTCCCCTGTTAAAGC
*Apkcι*	CAGGCTGTACGAGCTGAACAAG	GGAAATACATGAATCAAGAGTTCAGAATC
*pard3*	CCCAACTTTTCCCTCGATGATA	AACAACCCCAATGTTCTGCC
*pard3* (to show KO)	GAGATATTCGGCAGTGAGCTG	GAAGGCGTGACCTCAATTTCA

### Genotyping

Mice were genotyped from genomic DNA isolated from tail biopsies. To show deletion of the floxed allele, DNA was isolated from FACS sorted erythroid cells (Ter119 positive). *EpoR-Cre* WT/Tg alleles were detected using the following primers 5’-TCCCGGACCCCAAGTTTGAG-3’, 5’-AAGCCTCTGCCCTGAGCATCAC-3’ and 5’-GTGTGGCTGCCCCTTCTGCCA-3’ [27]. The PCR program employed was 95°C for 5 minutes, followed by 43 cycles of 95°C for 30seconds, 62°C for 30seconds, 72°C for 30seconds, and a final elongation step at 72°C for 5 minutes. *Pard3* WT/Floxed alleles were detected using the following primers 5’-AGGCTAGCCTGGGTGATTTGAGACC-3’ and 5’-TTCCCTGAGGCCTGACACTCCAGTC-3’. The PCR reaction was performed at 95°C for 5 minutes, followed by 35 cycles of 95°C for 30 seconds, 64°C for 30 seconds and 72°C for 30 seconds. A final elongation was performed at 72°C for 5 minutes. The recombined *Pard3* allele was detected using the following primers 5’-TACCGTTAACTGCAGCTCGGCTCTG-3’ and 5’-AGCTGGCGCTGGTACCATCTCCTCC-3’. The PCR program employed was 94°C for 3 minutes, followed by 40 cycles of 94°C for 30 seconds, 64°C for 60 seconds, 72°C for 45 seconds. A final elongation was performed at 72°C for 3minutes.

WT and LoxP-flanked *Scrib* alleles were detected using the following primer sequences 5’-GCCATGGTGGCAGAGGTTGG-3’ and 5’-TGCTTTCTCCCAGACTCAGG-3’. The recombined *Scrib* allele was detected using the following primer sequence 5’-GAGAAAGTTGGGCCTCAGTG-3’. The PCR program employed was 95°C for 2 minutes, followed by 35 cycles of 94°C for 30 seconds, 56°C for 30 seconds, 72°C for 30 seconds, and an elongation step of 72°C for 10 minutes.

Gpsm2 WT and deleted *Gpsm2* alleles were detected using the following primer sequences 5’-TCCTCCATCTGCTGCCACTAAG-3’, 5’-ACAGCCACCGAAGGTCACAAAG-3’ and 5’-TTCAGTAGGTTACCACACCATCCTG-3’. The PCR program employed was 95°C for 5 minutes, followed by 43 cycles of 95°C for 30 seconds, 66°C for 30 seconds and 72°C for 1 minute, and an elongation step of 72°C for 5 minutes.

### Osmotic fragility test

1μl of blood (from eye bleeds) was added to 200μl of various NaCl concentrations (1, 0.85, 0.75, 0.65, 0.6, 0.55, 0.5, 0.45, 0.4, 0.35, 0.3, 0.2, 0.1, 0% NaCl in dH_2_O). The blood was gently mixed with the NaCl solution and incubated at room temperature for 30 minutes. The sample was gently mixed again and then spun in a microcentrifuge at 14.000rpm for 2 minutes. The supernatant was transferred into a well of a 96-well plate and absorption measured (OD 550nm) by a plate reader. Percent hemolysis was calculated by dividing the absorption measured for the sample by the absorption measured in the well containing that sample in 0% NaCl (max. hemolysis), and by subsequently multiplying the quotient by 100.

## Results and Discussion

### Late stage erythroblasts express polarity regulators

To test whether the core conserved ACD machinery was involved in erythroid enucleation, we first examined the expression of ACD regulators in terminal differentiating erythroblasts and found that orthochromatic erythroblasts (the stage prior to enucleation) express genes of the Scribble complex (*Scrib*, *Lgl1*, *Lgl2*, *Dlg1*, *Dlg3*, *Dlg4*), the Pins complex (*Gpsm2*, *Insc*), and the Par3 complex (*aPKCζ*, *aPKCi*, *Pard3*) (S1 Fig). Of all genes surveyed, *Pard3* was significantly upregulated in orthochromatic erythroblasts compared to earlier, proliferating stages (S1C Fig).

### Characterization of knockout mouse models

We next tested if Par3, and other ACD regulators, Scribble and Gpsm2, play a role in erythroid enucleation. Par3 deficiency in the whole mouse results in defective cardiac development and embryonic lethality in the midgestational stage[22]. Similarly, Scribble null mice are neonatal lethal due to neural tube and abdominal wall closure defects[28, 29]. To investigate the biological functions of these polarity regulators in erythroid cells *in vivo*, mice harboring conditional *Pard3*[22] or *Scrib*[23] alleles were crossed to *Epor*-Cre^ki/+^ mice[21]. Expression of Cre recombinase from the endogenous *Epor* locus targets Cre expression to erythroid cells and therefore depletes Par3/Scribble specifically in the erythroid compartment as confirmed by PCR and qPCR analysis (S2A and S2B Fig). To assess Gpsm2 loss of function, we utilized *Gpsm2*^ΔC^ mice that carry a germline intragenic deletion of the GoLoco motif[24]. We confirmed the presence of this deletion by PCR and qPCR analysis (S2C Fig). Defective asymmetric cell divisions have been observed in these mice previously during neurogenesis[24].

### Polarity regulators Par3, scribble and Gpsm2 are not essential for red blood cell development

**Fig 1:**
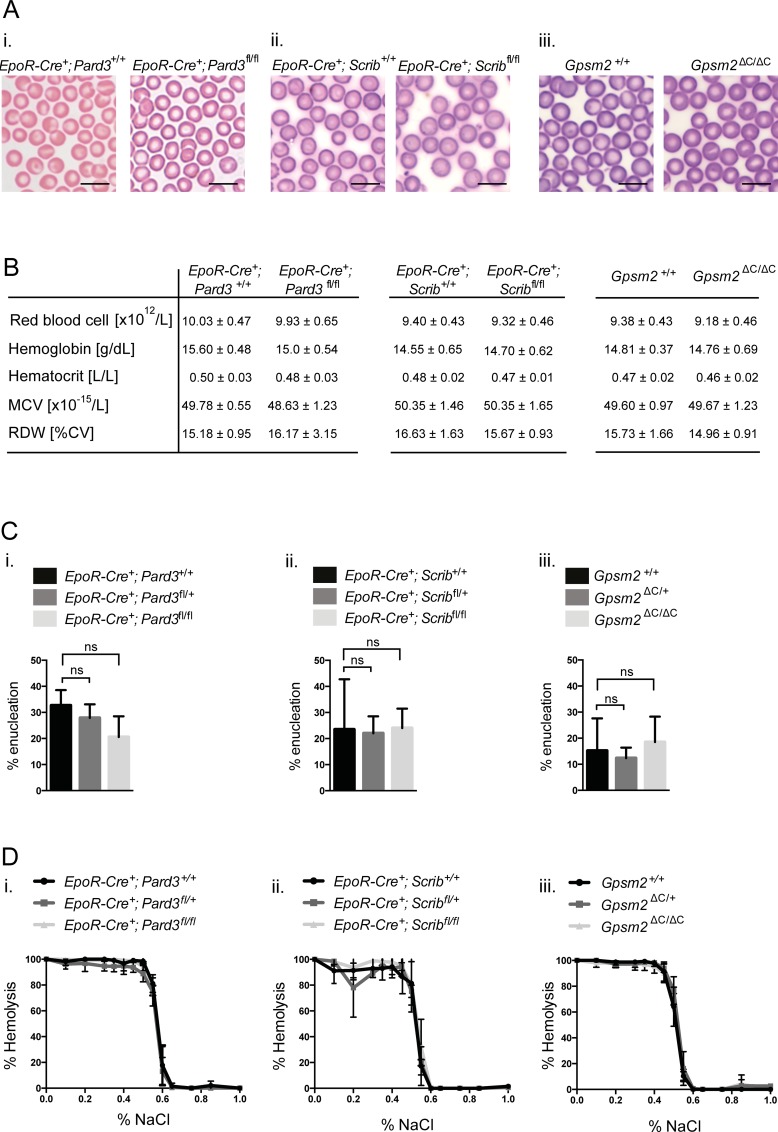
The ACD regulators Par3, Scribble and Gpsm2 are not required for erythroid enucleation during steady-state erythropoiesis. **(A)** Representative images of blood smears from peripheral blood derived from age-matched mice of the indicated genotypes of the different mouse models. Scale bar = 10μm. **(B)** Whole blood analysis using the CELL-DYN Sapphire System was performed on peripheral blood derived from age-matched mice of the indicated genotypes of the indicated mouse models. Data represent the mean (+/- SD) of 3–7 independent mice. **(C)** Orthochromatic erythroblasts were enriched by FACS (Aria II) from bone marrow of mice of the indicated genotypes of the indicated mouse models and incubated in 96-well plates at 30.000 cells per well. Graphs showing enucleation efficiencies 5h post sort quantified by LSR II. Data represent the mean (+/- SD) of 3–4 independent experiments. *P< 0.05, **P< 0.01, ***P< 0.001, ****P< 0.0001 (unpaired student’s t-test). **(D)** Whole blood derived from aged-matched mice of the indicated genotypes of the different mouse models was exposed to decreasing concentrations of sodium chloride (NaCl) and the degree of hemolysis measured colorimetrically. Data represent the mean (+/- SD) of 3–5 independent experiments.

Investigation of blood smears of peripheral blood collected from age-matched mice of the indicated genotypes revealed no enucleation defects in red blood cells in the absence of functional erythroid Par3, Scribble or Gpsm2 expression (Fig 1A (i-iii)). Whole blood analysis also confirmed no significant changes in other parameters including hemoglobin, hematocrit, mean corpuscular volume and red cell distribution width in mice with erythroid cells deficient for Par3, Scribble or Gpsm2 (Fig 1B (i-iii)). These results suggested that the key ACD regulators may not be required for erythroid enucleation *in vivo*. To formally exclude the possibility that a redundant physiological mechanism might act to rescue ACD defects *in vivo*, we investigated the ability of Par3, Scribble or Gpsm2-depleted orthochromatic erythroblasts to enucleate in media *ex vivo*[26] in the absence of surrounding supporting microenvironment. We found no differences in enucleation efficiencies of Par3, Scribble or Gpsm2 depleted erythroblasts compared to age-matched wild-type counterparts isolated from bone marrow (Fig 1C), indicating that the key ACD regulators Par3, Gpsm2 or Scribble are not essential for enucleation.

Osmotic fragility is affected in erythrocytes with abnormal membrane integrity and/or abnormal surface area-to-volume ratios[30]. As many membrane proteins are asymmetrically distributed during the enucleation event, we also investigated the membrane integrity of erythrocytes depleted of Par3, Scribble or Gpsm2 by measuring osmotic fragility. However, no difference in the degree of hemolysis could be identified (Fig 1D (i-iii)).

**Fig 2:**
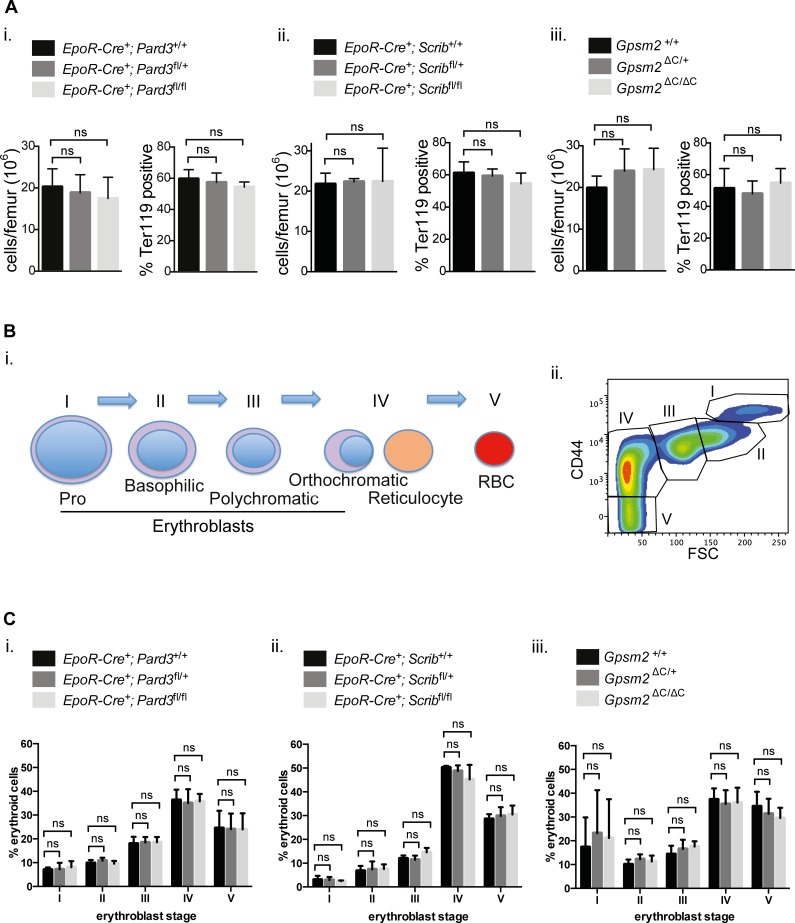
The ACD regulators Par3, Scribble or Gpsm2 are not required for steady-state erythropoiesis in the bone marrow. **(A)** Graphs showing total cell numbers per femur (on left) and percentages of erythroid cells (Ter119 positive) in femurs (on right) isolated from age-matched mice of the indicated genotypes of the different mouse models. Data are mean (+/- SD) of 3–6 independent experiments. **(B)** (i.) Diagram showing the distinct erythroid populations at the different maturation stages (I-V) that can be identified by FACS. (ii.) Representative FACS plot showing erythroid differentiation stages in the bone marrow. **(C)** Bar graphs showing percentages of erythroid cells at the different developmental stages (I-V) during homeostasis in the bone marrow harvested from age-matched mice of the indicated genotypes of the different mouse models. Data represent the mean (+/- SD) of 3–6 independent experiments. *P< 0.05, **P< 0.01, ***P< 0.001, ****P< 0.0001 (unpaired student’s t-test).

To exclude the possibility that long-term physiological compensation in erythropoiesis was not masking enucleation defects, we investigated whether erythropoiesis in general is affected in animals harboring erythroblasts that lack ACD regulators. Analysis of bone marrow cellularity (Fig 2A, left) and the proportion of erythroid cells (Fig 2A, right) in femoral bone marrow, revealed no significant differences between mice deficient of erythroid Par3, Scribble or Gpsm2 expression compared to age-matched wild-type controls. Analysis of erythropoiesis during homeostasis in bone marrow also showed no significant difference in the percentage of the different developmental erythroblast stages (Fig 2B and 2C). Altogether, these data indicate that the Par3, Scribble and Gpsm2 polarity regulators are dispensable for the full maturation of erythrocytes *in vivo*.

### Polarity regulators Par3, scribble and Gpsm2 are not essential for red blood cell development during stress erythropoiesis

**Fig 3:**
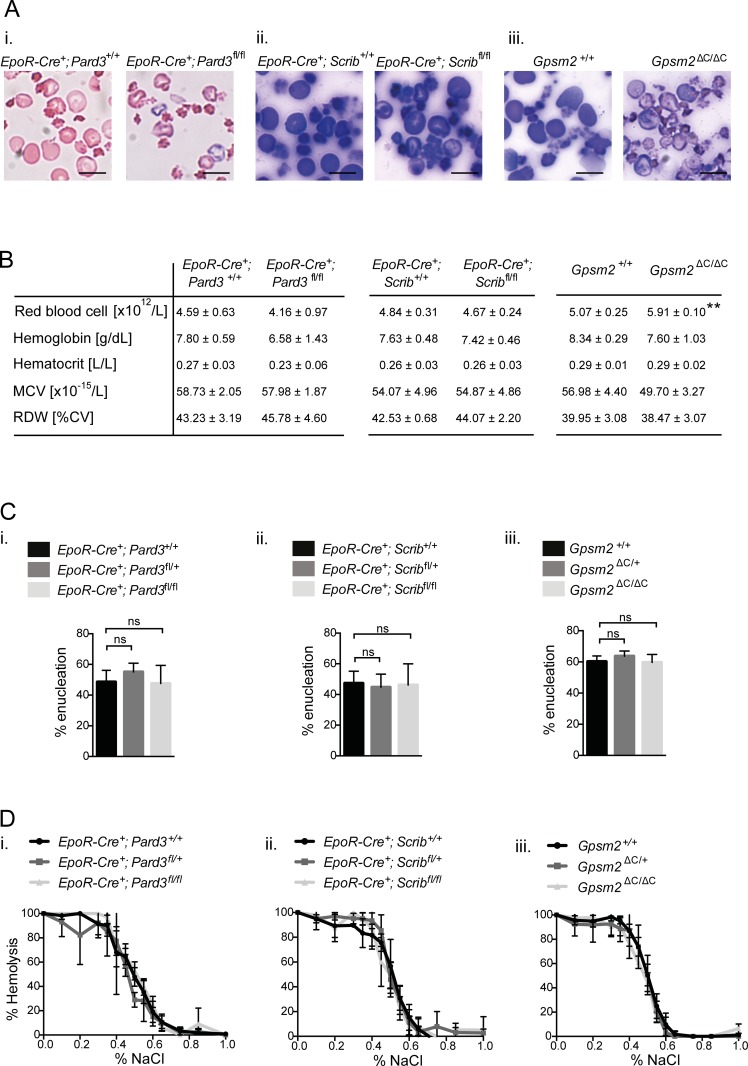
The ACD regulators Par3, Scribble and Gpsm2 are not required for erythroid enucleation during stress erythropoiesis. **(A)** Representative images of blood smears from peripheral blood derived from PHZ treated, age-matched mice of the indicated genotypes of the different mouse models. Scale bar = 10μm. **(B)** Whole blood analysis using the CELL-DYN Sapphire System was performed on peripheral blood derived from PHZ treated, age-matched mice of the indicated genotypes of the indicated mouse models. Data represent the mean (+/- SD) of 3–6 independent experiments. **(C)** Orthochromatic erythroblasts were enriched by FACS (Aria II) from spleen of PHZ treated mice of the indicated genotypes of the indicated mouse models and incubated in 96-well plates at 30000 cells per well. Graphs showing enucleation efficiencies 5h post sort quantified by LSR II. Data represent the mean (+/- SD) of 3 independent experiments. *P< 0.05, **P< 0.01, ***P< 0.001, ****P< 0.0001 (unpaired student’s t-test). **(D)** Whole blood derived from PHZ treated, aged-matched mice of the indicated genotypes of the different mouse models was exposed to decreasing concentrations of sodium chloride (NaCl) and the degree of hemolysis measured colorimetrically. Data represent the mean (+/- SD) of 3 independent experiments.

As we did not observe any enucleation deficits in erythroblasts depleted of functional Par3, Scribble or Gpsm2 during steady-state conditions, we next evaluated whether loss of these ACD regulators impacted on enucleation during stress erythropoiesis as this could potentially reveal rate-limiting requirements for these genes in enucleation. However, no enucleation defects were observed in the different mouse models based on blood smear analysis (Fig 3A (i-iii)) and whole blood analysis (Fig 3B (i-iii)) following phenylhydrazine (PHZ) -induced hemolytic stress erythropoiesis. We did observe a small but significant increase in the number of erythrocytes in the peripheral blood of *Gpsm2*^ΔC/ΔC^ mice, compared to age-matched controls but no other differences were observed (Fig 3A).

To test whether the *ex vivo* enucleating efficiency of orthochromatic erythroblasts isolated from spleens during stress erythropoiesis was affected by the functional loss of Par3, Scribble or Gpsm2, we compared enucleation rates of splenic orthochromatic erythroblasts from PHZ treated mice of the indicated genotypes to those of their age-matched controls (Fig 3C (i-iii)). Interestingly, the basal enucleation rate is much higher in splenic orthochromatic erythroblasts following PHZ induced stress erythropoiesis (around 60%) compared to the enucleation rate of orthochromatic erythroblasts isolated from bone marrow (up to 30%) (compare Fig 1C to Fig 3C). Regardless, no differences in enucleation efficiencies were observed between genotypes, suggesting that Par3, Scribble or Gpsm2 are not required for enucleation in the spleen during stress erythropoiesis. In addition, peripheral blood from PHZ treated mice lacking erythroid expression of Par3 or Scribble or whole body Gpsm2 were subjected to decreasing concentrations of NaCl and the degree of hemolysis measured and compared to age-matched controls. Again, no difference in the degree of hemolysis could be identified (Fig 3D).

**Fig 4:**
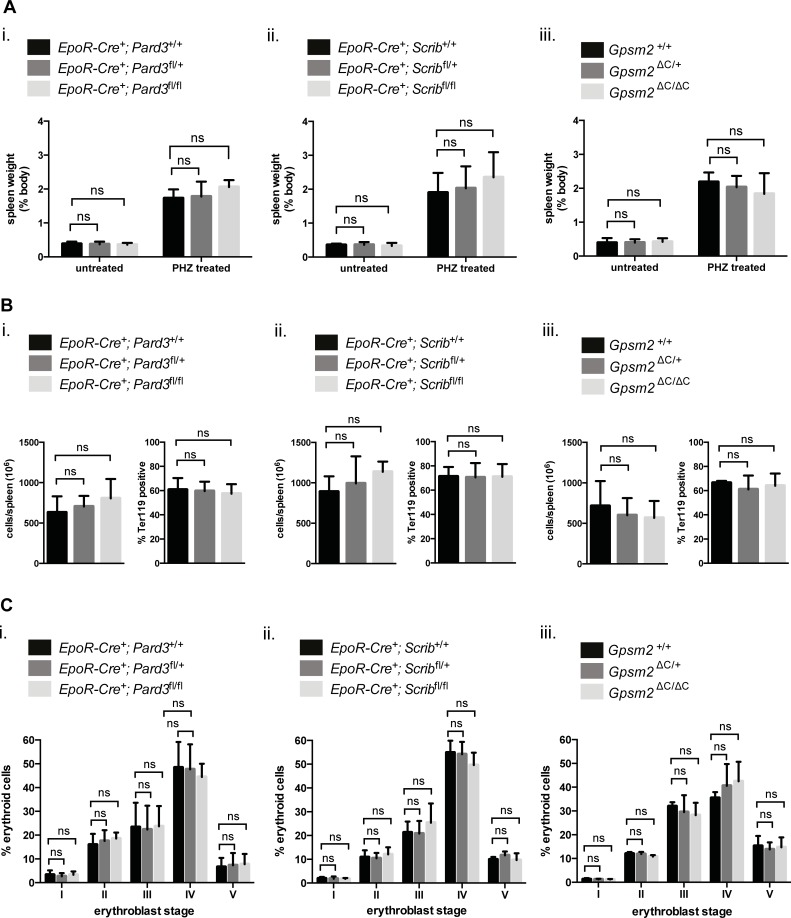
The ACD regulators Par3, Scribble and Gpsm2 are not required for stress erythropoiesis in the spleen. **(A)** Graphs showing spleen weights relative to body weights determined from untreated and PHZ treated, age-matched mice of the indicated genotypes of the different mouse models. Data represent the means (+/- SD) of 3–5 independent experiments. **(B)** Graphs showing total cell numbers per spleen (on left) and percentages of erythroid cells (Ter119 positive) in spleen (on right) isolated from PHZ treated, age-matched mice of the indicated genotypes of the different mouse models. Data represent the means (+/- SD) of 3–6 independent experiments. **(C)** Bar graphs showing percentages of erythroid cells at the different developmental stages (I-V) during stress erythropoiesis in the spleen from PHZ treated mice of the indicated genotypes of the different mouse models. Data represent the means (+/- SD) of 3–5 independent experiments. *P< 0.05, **P< 0.01, ***P< 0.001, ****P< 0.0001 (unpaired student’s t-test).

Analysis of stress erythropoiesis in general also showed no defects: Spleen weights revealed no significant differences in mice deficient of erythroid Par3, Scribble or Gpsm2 expression compared to age-matched wild-type controls (Fig 4A). Analysis of cellularity (Fig 4B, left) and the proportion of erythroid cells (Fig 4A, right) or their erythroid subsets (Fig 4C) in suspensions made from spleens isolated from PHZ treated mice, also revealed no significant differences. Collectively, these results suggest that enucleation is not affected by erythoid specific loss of functional Par3 or Scribble, or whole body loss of Gpsm2 under steady-state or stress conditions.

It is important to note that efficient erythropoiesis is crucial for organismal survival and that knockdown of functionally important genes is often associated with either embryonic death or high redundancy. We have started to examine compensatory mechanisms by qPCR (S3 Fig) but could not detect any significant compensatory upregulation in the knockout models tested here at a transcriptional level. However, it will be crucial to test whether other ACD regulators can compensate for the loss of Par3, Scribble or Gpsm2 in erythroid cells. The generation and analysis of double knockout mutants may shed light on this possibility. Future experiments will also involve examining expression and localization of ACD regulators at the protein level. Nevertheless, given that Par3, Scribble and Gpsm2 are fundamental regulators of polarity and ACD in invertebrates and mammalian cells, our results strongly indicate that the extreme asymmetry during erythroid enucleation is not established through these key ACD regulators. Red blood cells develop in erythroblastic islands, where erythroblasts differentiate bound to a central macrophage[31]. It is therefore possible that adhesion to the extracellular environment, e.g. Emp (erythroblasts macrophage protein) dependent adhesion between erythroblasts and macrophages[32, 33], or adhesion between erythroblasts and fibronectin[34], or possibly adhesion amongst erythroblasts[35], could provide the necessary cues to control asymmetric positioning of the cytoskeleton prior and during enucleation, rather than cell-intrinsic asymmetry. The observation that erythroid enucleation can occur *in vitro* in the absence of many of these microenvironmental cues though also suggests that other non-deterministic and stochastic mechanisms could be utilized to generate these asymmetries. Indeed, a key difference between ACD and erythroid enculeation is that canonical ACD involves not just polarization, but also alignment of that polarity with the spindle. Further, instead of a specific directionality, the recruitment of the nucleus to any part of the erythrocyte cortex might be the only requirement. Together, our findings suggest that erythrocyte enucleation may only have a minimal requirement for extracellular cues and the coordination provided by polarity complexes.

## Supporting Information

S1 Fig ACD genes are expressed in orthochromatic erythroblasts. Gene expression of genes from Scribble **(A)**, Gpsm2 **(B)** and Par **(C)** complex in orthochromatic compared to late, but still proliferating erythroblasts isolated from spleens of phenylhydrazine (PHZ) treated wild-type mice. Data are means (+/- SD) of 3 independent experiments. *P< 0.05, **P< 0.01, ***P< 0.001, ****P< 0.0001 (unpaired student’s t-test).
(TIF)

S2 Fig Molecular characterization of mouse models. **(A)** To investigate the biological functions of polarity regulator Par3 *in vivo*, mice harboring a *Pard3* construct flanked by loxP sites were crossed to *EpoR*-*Cre*^+^ mice. (i.) Representative PCR analysis showing deletion of the floxed *Pard3* allele in erythroblasts isolated by FACS (Aria II) from bone marrow (BM) suspensions harvested from mice of the indicated genotypes (ii.) Graph showing relative *Pard3* gene expression measured in FACS sorted erythroid cells derived from bone marrow harvested from mice of the indicated genotypes. Data represent the mean (+/- SD) of 3 independent experiments. **(B)** Mice harboring a *Scrib* construct flanked by loxP sites were crossed to *EpoR*-*Cre*^+^ mice. (i.) Representative PCR analysis showing deletion of the floxed *Scrib* allele in erythroblasts isolated by FACS (Aria II) from bone marrow (BM) suspensions harvested from mice of the indicated genotypes (ii.) Graph showing relative *Scrib* gene expression measured in FACS sorted erythroid cells derived from bone marrow harvested from mice of the indicated genotypes. Data represent the mean (+/- SD) of 2–3 independent experiments. **(C)** Characterization of Gpsm2^ΔC^ mice. (i.) Representative PCR analysis showing deletion of the *Gpsm2* allele in erythroblasts isolated by FACS (Aria II) from bone marrow (BM) suspensions harvested from mice of the indicated genotypes (ii.) Graph showing relative *Gpsm2* gene expression measured in FACS sorted erythroid cells derived from bone marrow harvested from mice of the indicated genotypes. Data represent the mean (+/- SD) of 3 independent experiments. *P< 0.05, **P< 0.01, ***P< 0.001, ****P< 0.0001 (unpaired student’s t-test).
(TIF)

S3 Fig Characterization of potential compensatory mechanisms. Gene expression of genes from Scribble, Gpsm2 and Par complex in erythroblasts isolated by FACS (Aria II) from bone marrow suspensions harvested from **(A)** *EpoR*-*Cre*^+^;*Pard3*^fl/fl^, **(B)** *EpoR*-*Cre*^+^;*Scrib*^fl/fl^ and **(C)** *Gpsm2*^ΔC^ mice and their age-matched controls. Data are means (+/- SD) of 3 independent experiments. *P< 0.05, **P< 0.01, ***P< 0.001, ****P< 0.0001 (unpaired student’s t-test).
(TIF)

## References

[1] Keerthivasan G, Wickrema A, Crispino JD. Erythroblast enucleation. Stem cells international. 2011;2011:139851 Epub 2011/10/19. PubMed Central PMCID: PMC3189604. 10.4061/2011/139851 22007239 PMC3189604

[2] Ji P, Murata-Hori M, Lodish HF. Formation of mammalian erythrocytes: chromatin condensation and enucleation. Trends in cell biology. 2011;21(7):409–15. Epub 2011/05/20. PubMed Central PMCID: PMC3134284. 10.1016/j.tcb.2011.04.003 21592797 PMC3134284

[3] Migliaccio AR. Erythroblast enucleation. Haematologica. 2010;95(12):1985–8. Epub 2010/12/03. PubMed Central PMCID: PMC2995553. 10.3324/haematol.2010.033225 21123437 PMC2995553

[4] Hebiguchi M, Hirokawa M, Guo YM, Saito K, Wakui H, Komatsuda A, et al Dynamics of human erythroblast enucleation. International journal of hematology. 2008;88(5):498–507. Epub 2008/12/02. 10.1007/s12185-008-0200-6 19043811

[5] Wang J, Ramirez T, Ji P, Jayapal SR, Lodish HF, Murata-Hori M. Mammalian erythroblast enucleation requires PI3K-dependent cell polarization. Journal of cell science. 2012;125(Pt 2):340–9. Epub 2012/02/15. PubMed Central PMCID: PMC3283871. 10.1242/jcs.088286 22331356 PMC3283871

[6] Ubukawa K, Guo YM, Takahashi M, Hirokawa M, Michishita Y, Nara M, et al Enucleation of human erythroblasts involves non-muscle myosin IIB. Blood. 2012;119(4):1036–44. Epub 2011/11/04. PubMed Central PMCID: PMC3352306. 10.1182/blood-2011-06-361907 22049517 PMC3352306

[7] Koury ST, Koury MJ, Bondurant MC. Cytoskeletal distribution and function during the maturation and enucleation of mammalian erythroblasts. The Journal of cell biology. 1989;109(6 Pt 1):3005–13. Epub 1989/12/01. PubMed Central PMCID: PMC2115945. 2574178 10.1083/jcb.109.6.3005 PMC2115945

[8] Yoshida H, Kawane K, Koike M, Mori Y, Uchiyama Y, Nagata S. Phosphatidylserine-dependent engulfment by macrophages of nuclei from erythroid precursor cells. Nature. 2005;437(7059):754–8. Epub 2005/09/30. 10.1038/nature03964 16193055

[9] Ji P, Jayapal SR, Lodish HF. Enucleation of cultured mouse fetal erythroblasts requires Rac GTPases and mDia2. Nature cell biology. 2008;10(3):314–21. Epub 2008/02/12. 10.1038/ncb1693 18264091

[10] Lee JC, Gimm JA, Lo AJ, Koury MJ, Krauss SW, Mohandas N, et al Mechanism of protein sorting during erythroblast enucleation: role of cytoskeletal connectivity. Blood. 2004;103(5):1912–9. Epub 2003/10/18. 10.1182/blood-2003-03-0928 14563645

[11] Knoblich JA. Asymmetric cell division: recent developments and their implications for tumour biology. Nature reviews Molecular cell biology. 2010;11(12):849–60. Epub 2010/11/26. 10.1038/nrm3010 21102610 PMC3941022

[12] Bilder D, Li M, Perrimon N. Cooperative regulation of cell polarity and growth by Drosophila tumor suppressors. Science. 2000;289(5476):113–6. Epub 2000/07/07. 10884224 10.1126/science.289.5476.113

[13] Siller KH, Cabernard C, Doe CQ. The NuMA-related Mud protein binds Pins and regulates spindle orientation in Drosophila neuroblasts. Nature cell biology. 2006;8(6):594–600. Epub 2006/05/02. 10.1038/ncb1412 16648843

[14] Yu F, Kuo CT, Jan YN. Drosophila neuroblast asymmetric cell division: recent advances and implications for stem cell biology. Neuron. 2006;51(1):13–20. Epub 2006/07/04. 10.1016/j.neuron.2006.06.016 16815328

[15] Duncan FE, Moss SB, Schultz RM, Williams CJ. PAR-3 defines a central subdomain of the cortical actin cap in mouse eggs. Developmental biology. 2005;280(1):38–47. Epub 2005/03/16. 10.1016/j.ydbio.2004.12.034 15766746

[16] Pham K, Sacirbegovic F, Russell SM. Polarized cells, polarized views: asymmetric cell division in hematopoietic cells. Frontiers in immunology. 2014;5:26 Epub 2014/02/20. PubMed Central PMCID: PMC3909886. 10.3389/fimmu.2014.00026 24550912 PMC3909886

[17] Sugimoto M, Yasuda T. Asymmetric (differential) cell division of thymic lymphocytes by means of cytoplasmic polarization: possible biological meanings. Thymus. 1983;5(5–6):297–310. Epub 1983/09/01. 6606874

[18] Yassin M, Russell SM. Polarity and asymmetric cell division in the control of lymphocyte fate decisions and function. Current opinion in immunology. 2016;39:143–9. Epub 2016/03/06. 10.1016/j.coi.2016.02.004 26945468

[19] Ting SB, Deneault E, Hope K, Cellot S, Chagraoui J, Mayotte N, et al Asymmetric segregation and self-renewal of hematopoietic stem and progenitor cells with endocytic Ap2a2. Blood. 2012;119(11):2510–22. Epub 2011/12/17. 10.1182/blood-2011-11-393272 22174158

[20] Neumuller RA, Knoblich JA. Dividing cellular asymmetry: asymmetric cell division and its implications for stem cells and cancer. Genes & development. 2009;23(23):2675–99. Epub 2009/12/03. PubMed Central PMCID: PMC2788323. 19952104 10.1101/gad.1850809 PMC2788323

[21] Singbrant S, Russell MR, Jovic T, Liddicoat B, Izon DJ, Purton LE, et al Erythropoietin couples erythropoiesis, B-lymphopoiesis, and bone homeostasis within the bone marrow microenvironment. Blood. 2011;117(21):5631–42. Epub 2011/03/23. 10.1182/blood-2010-11-320564 21421837

[22] Hirose T, Karasawa M, Sugitani Y, Fujisawa M, Akimoto K, Ohno S, et al PAR3 is essential for cyst-mediated epicardial development by establishing apical cortical domains. Development. 2006;133(7):1389–98. Epub 2006/03/03. 10.1242/dev.02294 16510507

[23] Pearson HB, Perez-Mancera PA, Dow LE, Ryan A, Tennstedt P, Bogani D, et al SCRIB expression is deregulated in human prostate cancer, and its deficiency in mice promotes prostate neoplasia. The Journal of clinical investigation. 2011;121(11):4257–67. Epub 2011/10/04. PubMed Central PMCID: PMC3223862. 10.1172/JCI58509 21965329 PMC3223862

[24] Konno D, Shioi G, Shitamukai A, Mori A, Kiyonari H, Miyata T, et al Neuroepithelial progenitors undergo LGN-dependent planar divisions to maintain self-renewability during mammalian neurogenesis. Nature cell biology. 2008;10(1):93–101. Epub 2007/12/18. 10.1038/ncb1673 18084280

[25] Chen K, Liu J, Heck S, Chasis JA, An X, Mohandas N. Resolving the distinct stages in erythroid differentiation based on dynamic changes in membrane protein expression during erythropoiesis. Proceedings of the National Academy of Sciences of the United States of America. 2009;106(41):17413–8. Epub 2009/10/07. PubMed Central PMCID: PMC2762680. 10.1073/pnas.0909296106 19805084 PMC2762680

[26] Wolwer CB, Pase LB, Pearson HB, Godde NJ, Lackovic K, Huang DC, et al A Chemical Screening Approach to Identify Novel Key Mediators of Erythroid Enucleation. PloS one. 2015;10(11):e0142655 Epub 2015/11/17. 10.1371/journal.pone.0142655 26569102 PMC4646491

[27] Heinrich AC, Pelanda R, Klingmuller U. A mouse model for visualization and conditional mutations in the erythroid lineage. Blood. 2004;104(3):659–66. Epub 2004/04/20. 10.1182/blood-2003-05-1442 15090451

[28] Murdoch JN, Rachel RA, Shah S, Beermann F, Stanier P, Mason CA, et al Circletail, a new mouse mutant with severe neural tube defects: chromosomal localization and interaction with the loop-tail mutation. Genomics. 2001;78(1–2):55–63. Epub 2001/11/15. 10.1006/geno.2001.6638 11707073

[29] Zarbalis K, May SR, Shen Y, Ekker M, Rubenstein JL, Peterson AS. A focused and efficient genetic screening strategy in the mouse: identification of mutations that disrupt cortical development. PLoS biology. 2004;2(8):E219 Epub 2004/08/18. PubMed Central PMCID: PMC509294. 10.1371/journal.pbio.0020219 15314648 PMC509294

[30] Emerson CP Jr., Shein SC, Ham TH, Fleming EM, Castle WB. Studies on the destruction of red blood cells. IX. Quantitative methods for determining the osmotic and mechanical fragility of red cells in the peripheral blood and splenic pulp; the mechanism of increased hemolysis in hereditary spherocytosis (congenital hemolytic jaundice) as related to the functions of the spleen. AMA archives of internal medicine. 1956;97(1):1–38. Epub 1956/01/01. 13275144 10.1001/archinte.1956.00250190017001

[31] Bessis M. [Erythroblastic island, functional unity of bone marrow]. Revue d'hematologie. 1958;13(1):8–11. Epub 1958/01/01. 13555228

[32] Hanspal M, Hanspal JS. The association of erythroblasts with macrophages promotes erythroid proliferation and maturation: a 30-kD heparin-binding protein is involved in this contact. Blood. 1994;84(10):3494–504. Epub 1994/11/15. 7949103

[33] Soni S, Bala S, Gwynn B, Sahr KE, Peters LL, Hanspal M. Absence of erythroblast macrophage protein (Emp) leads to failure of erythroblast nuclear extrusion. The Journal of biological chemistry. 2006;281(29):20181–9. Epub 2006/05/19. 10.1074/jbc.M603226200 16707498

[34] Patel VP, Lodish HF. A fibronectin matrix is required for differentiation of murine erythroleukemia cells into reticulocytes. The Journal of cell biology. 1987;105(6 Pt 2):3105–18. Epub 1987/12/01. PubMed Central PMCID: PMC2114745. 2961771 10.1083/jcb.105.6.3105 PMC2114745

[35] Choi HS, Lee EM, Kim HO, Park MI, Baek EJ. Autonomous control of terminal erythropoiesis via physical interactions among erythroid cells. Stem cell research. 2013;10(3):442–53. Epub 2013/03/19. 10.1016/j.scr.2013.02.003 23500644

