# Supplementary figures and images for: The Asymmetric Cell Division Regulators Par3, Scribble and Pins/Gpsm2 Are Not Essential for Erythroid Development or Enucleation
Source: PLoS One. 2017 Jan 17;12(1):e0170295. doi: 10.1371/journal.pone.0170295 (PMC5240992; doi:10.1371/journal.pone.0170295)

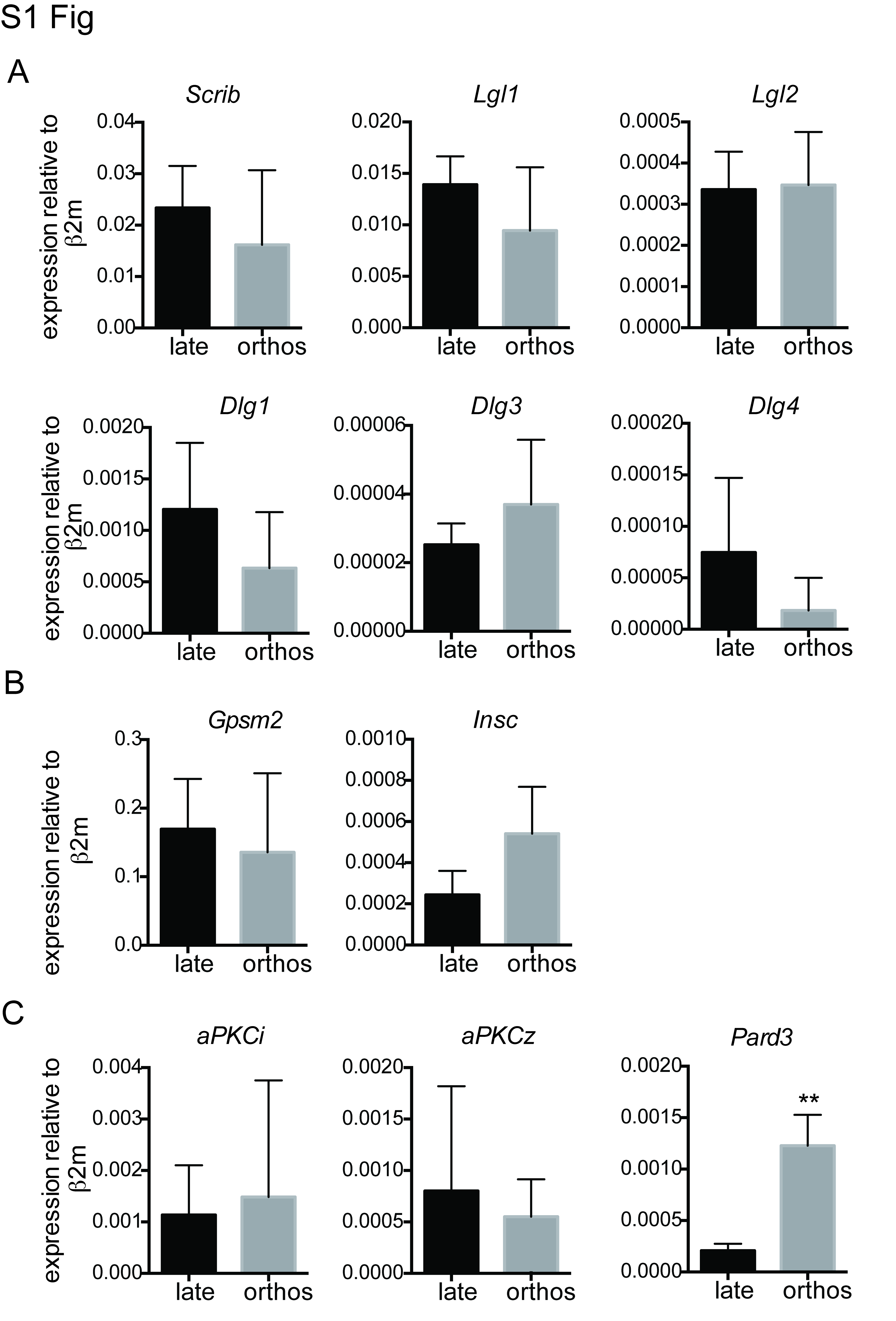

Supplement: S1 Fig — Gene expression of genes from Scribble (A), Gpsm2 (B) and Par (C) complex in orthochromatic compared to late, but still proliferating erythroblasts isolated from spleens of phenylhydrazine (PHZ) treated wild-type mice. Data are means (+/- SD) of 3 independent experiments. *P< 0.05, **P< 0.01, ***P< 0.001, ****P< 0.0001 (unpaired student’s t-test). (TIF) [file pone.0170295.s001.tif]

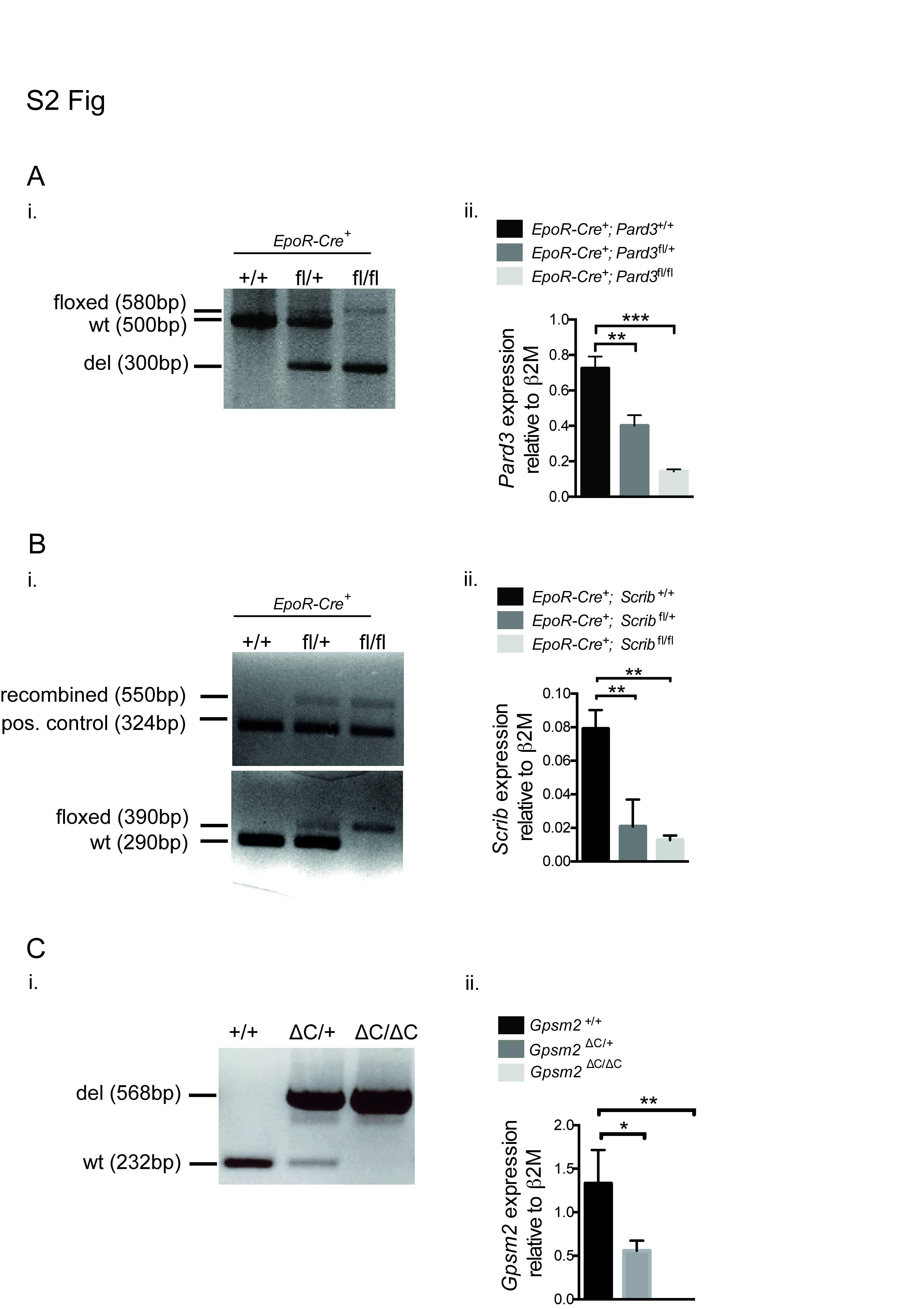

Supplement: S2 Fig — (A) To investigate the biological functions of polarity regulator Par3 in vivo, mice harboring a Pard3 construct flanked by loxP sites were crossed to EpoR-Cre+ mice. (i.) Representative PCR analysis showing deletion of the floxed Pard3 allele in erythroblasts isolated by FACS (Aria II) from bone marrow (BM) suspensions harvested from mice of the indicated genotypes (ii.) Graph showing relative Pard3 gene expression measured in FACS sorted erythroid cells derived from bone marrow harvested from mice of the indicated genotypes. Data represent the mean (+/- SD) of 3 independent experiments. (B) Mice harboring a Scrib construct flanked by loxP sites were crossed to EpoR-Cre+ mice. (i.) Representative PCR analysis showing deletion of the floxed Scrib allele in erythroblasts isolated by FACS (Aria II) from bone marrow (BM) suspensions harvested from mice of the indicated genotypes (ii.) Graph showing relative Scrib gene expression measured in FACS sorted erythroid cells derived from bone marrow harvested from mice of the indicated genotypes. Data represent the mean (+/- SD) of 2–3 independent experiments. (C) Characterization of Gpsm2ΔC mice. (i.) Representative PCR analysis showing deletion of the Gpsm2 allele in erythroblasts isolated by FACS (Aria II) from bone marrow (BM) suspensions harvested from mice of the indicated genotypes (ii.) Graph showing relative Gpsm2 gene expression measured in FACS sorted erythroid cells derived from bone marrow harvested from mice of the indicated genotypes. Data represent the mean (+/- SD) of 3 independent experiments. *P< 0.05, **P< 0.01, ***P< 0.001, ****P< 0.0001 (unpaired student’s t-test). (TIF) [file pone.0170295.s002.tif]

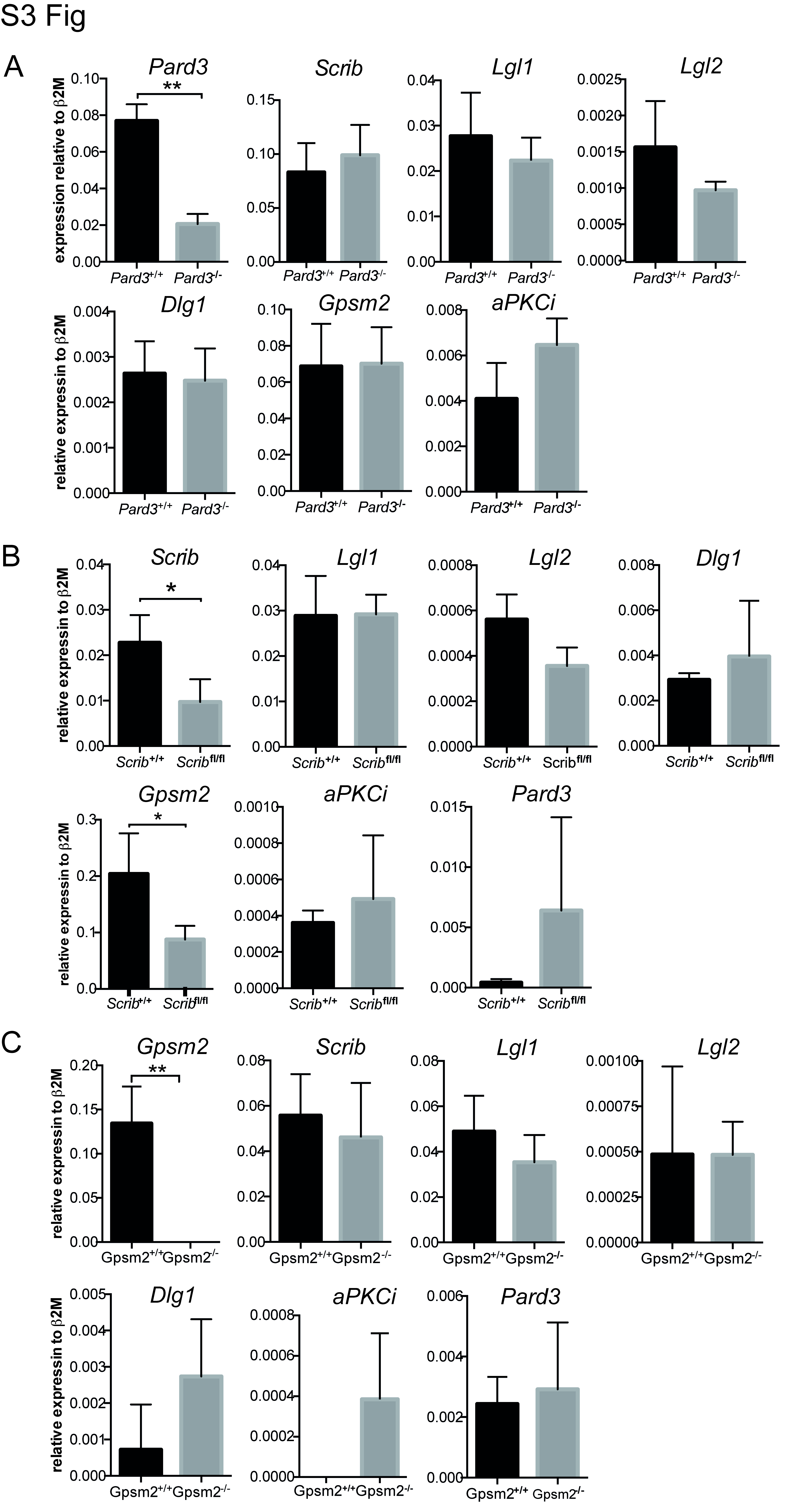

Supplement: S3 Fig — Gene expression of genes from Scribble, Gpsm2 and Par complex in erythroblasts isolated by FACS (Aria II) from bone marrow suspensions harvested from (A) EpoR-Cre+;Pard3fl/fl, (B) EpoR-Cre+;Scribfl/fl and (C) Gpsm2ΔC mice and their age-matched controls. Data are means (+/- SD) of 3 independent experiments. *P< 0.05, **P< 0.01, ***P< 0.001, ****P< 0.0001 (unpaired student’s t-test). (TIF) [file pone.0170295.s003.tif]
